# Development and Characterization of Bladder Cancer Patient-Derived Xenografts for Molecularly Guided Targeted Therapy

**DOI:** 10.1371/journal.pone.0134346

**Published:** 2015-08-13

**Authors:** Chong-xian Pan, Hongyong Zhang, Clifford G. Tepper, Tzu-yin Lin, Ryan R. Davis, James Keck, Paramita M. Ghosh, Parkash Gill, Susan Airhart, Carol Bult, David R. Gandara, Edison Liu, Ralph W. de Vere White

**Affiliations:** 1 Department of Internal Medicine, Division of Hematology/Oncology, University of California Davis, Sacramento, CA, 95817, United States of America; 2 Department of Urology, University of California Davis, Sacramento, CA, 95817, United States of America; 3 VA Northern California Health Care System, Mather, CA, 95655, United States of America; 4 Department of Biochemistry and Molecular Medicine, University of California Davis, Sacramento, CA, 95817, United States of America; 5 Department of Pathology and Laboratory Medicine, University of California Davis, Sacramento, CA, 95817, United States of America; 6 The Jackson Laboratory, Sacramento, CA, 95838, United States of America; 7 University of Southern California, Los Angeles, CA, 90089, United States of America; University of Michigan, UNITED STATES

## Abstract

**Background:**

The overarching goal of this project is to establish a patient-derived bladder cancer xenograft (PDX) platform, annotated with deep sequencing and patient clinical information, to accelerate the development of new treatment options for bladder cancer patients. Herein, we describe the creation, initial characterization and use of the platform for this purpose.

**Methods and Findings:**

Twenty-two PDXs with annotated clinical information were established from uncultured unselected clinical bladder cancer specimens in immunodeficient NSG mice. The morphological fidelity was maintained in PDXs. Whole exome sequencing revealed that PDXs and parental patient cancers shared 92–97% of genetic aberrations, including multiple druggable targets. For drug repurposing, an EGFR/HER2 dual inhibitor lapatinib was effective in PDX BL0440 (progression-free survival or PFS of 25.4 days versus 18.4 days in the control, p = 0.007), but not in PDX BL0269 (12 days versus 13 days in the control, p = 0.16) although both expressed *HER2*. To screen for the most effective MTT, we evaluated three drugs (lapatinib, ponatinib, and BEZ235) matched with aberrations in PDX BL0269; but only a PIK3CA inhibitor BEZ235 was effective (p<0.0001). To study the mechanisms of secondary resistance, a fibroblast growth factor receptor 3 inhibitor BGJ398 prolonged PFS of PDX BL0293 from 9.5 days of the control to 18.5 days (p<0.0001), and serial biopsies revealed that the MAPK/ERK and PIK3CA-AKT pathways were activated upon resistance. Inhibition of these pathways significantly prolonged PFS from 12 day of the control to 22 days (p = 0.001). To screen for effective chemotherapeutic drugs, four of the first six PDXs were sensitive to the cisplatin/gemcitabine combination, and chemoresistance to one drug could be overcome by the other drug.

**Conclusion:**

The PDX models described here show good correlation with the patient at the genomic level and known patient response to treatment. This supports further evaluation of the PDXs for their ability to accurately predict a patient’s response to new targeted and combination strategies for bladder cancer.

## Introduction

Bladder cancer is among the ten most common [1], yet the most understudied and underfunded, cancers [2]. There are two major groups of bladder cancer: non-myoinvasive and advanced cancer. Non-myoinvasive bladder cancer (NMIBC) accounts for 80% of cases at diagnosis. They are usually treated with transurethral resection and, in most cases, intravesical therapy. However, approximately 60% of patients recur at two years, and 25% progress to advanced stages [3–5]. It is those 20% cases with advanced stages at diagnosis and 25% of NMIBC cases that progressed to advanced stages that account for the high mortality of bladder cancer. Highly toxic platinum-based chemotherapy is commonly used in the treatment of advanced bladder cancer with a response rate of around 50% [6]. Even though cell lines are commonly used for preclinical studies, the correlation of drug sensitivity between cell lines and clinical trials is, in general, poor [7]. So far, no test is available to identify effective chemotherapy before administration of therapy. If patients are diagnosed with locally advanced bladder cancer without metastasis, radical cystectomy is usually performed that is associated with the worst health-related quality of life because of the surgery itself and associated post-operative complications [8,9]. In case of disease recurrence or prognosis, there is no standard second-line chemotherapy. There is no targeted therapy approved by the US Food and Drug Administration (FDA) even though recurrent genetic aberrations have been identified. There is no significant improvement in overall survival and prognosis over the last thirty years [10]. Therefore, there is a critical unmet need in bladder cancer to select effective first-line and salvage chemotherapy, and develop targeted therapy.

This project is to develop patient-derived xenografts (PDXs) to improve treatment outcomes of bladder cancer. Many animal models are currently used in cancer research, such as mouse xenografts developed from cancer cell lines and genetically engineered mouse models (GEMM). Even though these models have made tremendous contributions to cancer research, they fall short in meeting the individual patient-specific needs in the era of precision medicine. Recent developments in and convergence of cancer biology, “-omics” technologies, computational biology and drug development are revolutionizing targeted therapy and leading to a new level of “molecularly guided targeted therapy (MTT)”, meaning matching targeted therapy with patient-specific genetic aberrations in cancer. However, two recent clinical trials showed that matching MTT against patient-specific genetic aberrations was associated with disappointing response rates of 12% and 9%, respectively [11,12]. The low response rate of MTT is in part attributed to the fact that most cancers harbor multiple genetic aberrations [13–16], and that current computational biology technologies are unable to robustly distinguish driver mutations (those critical for cancer cell functions) from passenger mutations (those that have little functional consequence for cancer cells). We proposed that the patient-specific PDX platform developed here can not only potentially be used to screen for the most effective MTT to target the molecular drivers, and effective first-line and salvage chemotherapy, but also for drug repurposing and studying of secondary resistance mechanisms to guide further personalized therapy and drug development.

## Methods

### Development of patient-derived bladder cancer xenografts

The protocol to collect clinical information and cancer specimens from patients was approved by the University of California Davis Institutional Review Board (Protocol No. 218204). All participants provided written informed consent before participation in this study and before any specimens or clinical information was collected. The animal protocol was approved by the Jackson Laboratory (JAX) Institutional Animal Care and Use Committee (IACUC, Protocol No. 12027) and the UC Davis IACUC (Protocol No. 17794).

Fresh clinical cancer specimens (3–5mm^3^) were implanted subcutaneously into the flanks of 4–5 week old NOD.Cg-*Prkdc* ^*scid*^ *Il2rg* ^*tm1Wjl*^/SzJ (aka, NSG) mice. For each patient specimen, five NSG mice were implanted and monitored for tumor growth for up to five months. An orthotopic PDX model was generated via injection of single cell suspension from subcutaneous PDXs into the mouse bladder wall.

### RNA isolation

Hematoxylin and eosin stain was performed and the slides were reviewed to ensure that at least 85–90% of cells were cancer cells before specimen collection. Total cellular RNA was isolated from either fresh-frozen xenograft tumor pieces using the TRIzol Plus RNA Purification Kit (Life Technologies) or from formalin-fixed, paraffin-embedded (FFPE) specimens (8 x 12-μM sections) using the miRNeasy FFPE Kit (Qiagen), according to the manufacturer’s protocols. Total RNA was eluted from the columns in nuclease-free water and stored at -80°C. RNA concentration and purity were assessed with a NanoDrop 2000 Spectrophotometer (Thermo Scientific) and quality assessments (*e*.*g*., RNA integrity) were made using an Agilent 2100 Bioanalyzer (Agilent Technologies). For miRNA expression profiling, total cellular RNA (including the small RNA fraction) was isolated from FFPE bladder cancer specimens using the miRNeasy FFPE Kit (Qiagen) according to the manufacturer’s protocol.

### DNA isolation

DNA was isolated from fresh-frozen or FFPE patient tumor and xenograft samples (5 x 20-μM sections) using standard QIAamp DNA or QIAamp DNA FFPE Tissue Kits (Qiagen).

### Transcriptome profiling by RNA-Sequencing (RNA-Seq)

#### RNA-Seq library preparation and sequencing

Transcriptome profiling of P0 (passage 0) bladder cancer xenograft tumors was performed by RNA-Seq analysis. RNA-Sequencing (RNA-Seq) libraries were prepared from 1 μg total RNA using the TruSeq RNA Sample Preparation v2 Kit (Illumina, San Diego, CA) according to the manufacturer’s protocol. Briefly, poly-adenylated mRNA was purified from total RNA and ribosomal RNA removed by two rounds of binding to magnetic oligo-dT beads followed by RNA fragmentation, elution, and priming by incubation at 94°C for 8 minutes. Double-stranded cDNA was then generated by random-primed first-strand synthesis with SuperScript II reverse transcriptase and subsequent second strand synthesis with RNase H and DNA Polymerase I. The cDNA was blunt-ended with T4 and Klenow DNA polymerases to remove the 3′-overhangs and fill in 5′-overhangs, phosphorylated with T4 PNK, and then 3′-A tailed by incubation with Klenow Fragment (3´→5´ exo–) and dATP. Illumina paired-end (PE), indexed adapters were then ligated, followed by purification with AMPure XP beads. The library was then enriched by high-fidelity PCR amplification (15 cycles) and adapter-specific primers. The molar concentration of the libraries was determined by measuring concentration with a Qubit fluorometer (Invitrogen), determining the insert length with an Agilent 2100 Bioanalyzer, and then qPCR-based quantification (KAPA Library Quantification Kit). Libraries were submitted to the New York Genome Center for 100-bp paired-end, multiplex sequencing on a HiSeq 2000 sequencing system (8 libraries per lane; 2 lanes).

#### RNA-Seq data analysis

Image processing, base calling, quality scoring (Phred), and sample demultiplexing were executed by HiSeq Control Software with Real Time Analysis (HCS 1.5/RTA 1.13) and CASAVA 1.8 software (Illumina; San Diego, CA). Analysis of RNA-Seq data was performed using a standard TopHat-Cufflinks workflow [17]. Sequence reads (FASTQ format) were classified as human (graft) or mouse (host) using Xenome [18] and subsequently aligned to the reference human genome assembly (Feb. 2009, GRCh37/hg19) with TopHat, allowing for a maximum of two mismatches; TopHat utilizes the Bowtie aligner [19] and includes a tool for mapping splice junctions [20] for RNA-Seq read alignment to the reference human genome sequence (GRCh37/hg19). Gene- and transcript-level expression was comprehensively quantified with Cufflinks software [21] for *1)* transcript assembly, *2)* identification of splice variants, and 3) quantification of normalized expression as FPKM (fragments per kilobase of transcript per million mapped reads) values.

### Whole-exome sequencing (WES)

#### Preparation of whole exome-capture sequencing libraries and sequencing

DNA samples were prepared for whole-exome sequencing on the Illumina platform utilizing the SureSelect^XT^ Target Enrichment System (Agilent) in conjunction with the SureSelect^XT^ Human All Exon V4+UTRs capture library. This was performed according to the manufacturer’s protocols and proceeded in 3 general steps beginning with DNA fragmentation, followed by library preparation, and targeted enrichment for all exons and untranslated regions (UTRs). High-molecular weight DNA (3 μg) was sheared into fragments of mean peak size of 150–200 bp using a Covaris S220 focused-ultrasonicator and then purified using Agencourt AMPure XP magnetic beads. Standard protocols were utilized for adaptor ligation, indexing, high-fidelity PCR amplification. Subsequently, exome enrichment was performed by hybrid capture with the All Exon v4+UTRs capture library (789,141 biotinylated, ultra-long RNA oligomer baits) to capture the targeted sequences spanning 71Mb of the genome and encompassing of 20,965 genes and 334,378 exons. Capture libraries were amplified, pooled, and submitted to the New York Genome Center for 100-bp paired-end, multiplex sequencing on a HiSeq 2000 sequencing system (4 libraries per lane).

#### WES data analysis

Secondary analysis of the WES data consisted of read alignment to the reference genome sequence (GRCh37/hg19) using the Burrows-Wheeler Aligner (BWA) [22] and applying The Genome Analysis Toolkit (GATK) [23] for base quality score recalibration, indel realignment, duplicate removal, and performing SNV and INDEL discovery and genotyping across all samples simultaneously using standard hard filtering parameters or variant quality score recalibration [24]. Prior to alignment, reads were error-trimmed before the occurrence of a low-quality base (Phred score ≤20). In addition, for analysis of WES data derived from xenograft tissues, as well as patient tumor data used in comparisons, Xenome was utilized for human/mouse read classification and determination of levels of mouse genomic contamination [18]. Performance statistics for next-generation sequencing and subsequent analyses, including total numbers of reads, percentage mapping, and human/mouse read classification, are included in S1 Table and S2 Table.

Subsequent to the application of the GATK, variants were filtered for those having confirmed somatic mutation status and/or been identified as a somatic mutation in at least one tumor by using the complete Catalogue of Somatic Mutations in Cancer (COSMIC) and The Cancer Genome Atlas (TCGA) databases. In order to further define the likelihood of a previously confirmed somatic variant as being a somatic aberration in these PDX tumors, an additional filter was imposed to select for variant allele fractions in the range of 10–40% or 60–90%, thereby suggesting the presence of tumor heterogeneity and that the variant was derived from a tumor sub-population. Along these lines, several variants with “inferred somatic” status satisfied these criteria and were also included in the results. Although these do not correspond to an exact match in COSMIC or TCGA, filtering was performed with Ingenuity Variant Analysis (Qiagen, Inc.) to *1)* exclude variants that are associated with normal human genetic variation identified from large-scale sequencing projects, including the 1,000 Genomes Project, Complete Genomics Public Genomes, NHLBI GO Exome Sequencing Project (ESP), and dbSNP, and 2) to identify “non-dbSNP” variants with intermediate allele frequencies that would be characteristic of variants present in a heterogeneous tumor rather than in the germline.

### Efficacy study

This protocol was approved by the UC Davis Institutional Animal Care and Use Committee (IACUC, Protocol #17794) prior to study initiation. All the animal studies followed the IACUC guidelines. Female NSG mice at the age of 4–5 weeks were ordered from JAX, and were given at least one week to acclimate to the new environment before entering the study. To establish multiple PDXs to allow efficacy studies with multiple drugs, PDXs from Passage 2–4 were minced into 3–5 mm^3^ and injected into multiple mice either subcutaneously at the flank or orthotopically into the muscular layer of the bladder wall. When subcutaneous tumor sizes reached ~ 200 mm^3^, mice were treated with targeted therapeutic agents matched with the genetic alterations identified through deep sequencing as described above (**S1 Fig**). The following drugs were used in this study: sEphB4-HSA was developed through conjugation of soluble EphB4 to human serum albumin. It was provided by Parkash Gill, MD, at University of South California. Other drugs, including BGJ398 and BEZ235, were purchased from Selleck Chemicals (Houston, TX). For each treatment group, 8–10 mice were used to allow statistical analysis. Mice were monitored for tumor growth and alterations in clinical parameters, such as weight changes, waste elimination, coat texture, color, urine stains, crusting around the eyes, activity levels, and posture. Mice were sacrificed when the tumor size reached five times the baseline size (around 1,000 mm^3^). Several mice were sacrificed before treatment started or during treatment to determine the downstream signaling pathway activities using western blot, immunohistochemical staining, or immunofluorescence staining. Mice were sacrificed through pentobarbital overdose (180 mg/kg) or pentobarbital overdose (60 mg/kg) followed by cervical dislocation.

### Statistical analysis

The experiments were repeated at least in triplicate. Statistical analysis was performed by using GraphPad InStatTM software (GraphPad Software Inc., San Diego, CA) and ANOVA (Analysis Of Variance) was performed to compare the differences of the three treatment groups. All results were expressed as the mean ± standard error unless otherwise noted. A value of p<0.05 was considered statistically significant.

## Results

### Establishment of patient derived xenografts (PDX) from urothelial carcinoma

To date, we have established 22 PDXs from bladder cancer patients, representing 41% of the tumors implanted. Among these 22 PDXs, 13 PDXs were derived from advanced bladder cancer, and 9 PDXs from NMIBC. The clinical characteristics are shown in **Table 1**. The median age of the donor patients was 74 years (range: 54–83). Five of the 13 PDXs derived from advanced bladder cancer received prior neoadjuvant chemotherapy before implantation while none of the NMIBC did so.

**Table 1:** Clinical Characteristics of the donor patients. Twenty two PDXs were developed, including 13 from advanced bladder cancer and 9 from non-myoinvasive bladder cancer.

Stages	Tumor ID	Age (yrs)	Stage	Surgery	Prior chemo
**Myoinvasive bladder cancer**	BL0269F	58	pT4 N0 Mx	Cystectomy	No
	BL0293F	77	pT2a N2 Mx	Cystectomy	No
	BL0307F	78	pT3b N2 Mx	Cystectomy	No
	BL0382F	82	pT2 Nx Mx	TURBT	No
	BL0428F	70	pT2 Nx Mx	TURBT	No
	BL0429F	60	pT4a N3 M1	Cystectomy	No
	BL0479F	78	pT2b Nx Mx	Cystectomy	YES (carbo/gem/PTX)#
	BL0440F	71	pT4a N2 Mx	Cystectomy	YES (gem/cis)#
	BL0515F	78	pT3b N0 Mx	Cystectomy	YES (Gem/Cis)#
	BL0545F	70	pT2 N0 Mx	Cystectomy	No
	BL0601F	83	pT3 N0 Mx	Cystectomy	No
	BL0629F	74	pT3 N0 Mx	Cystectomy	No
	BL0645F	75	pT4a N2 Mx	Cystectomy	YES (MVAC)#
	BL0648	71	pT4a N2 Mx	Cystectomy	No. Adenocarcinoma
**Non-myo-invasive bladder cancer**	BL0262F	64	pTa High	TURBT	No
	BL0364F	76	pTa Low	TURBT	No
	BL0381F *	60	pTa High	TURBT	No
	BL0398F *	60	pT1 No Mx	Cystectomy	No
	BL0470F	55	pTa Nx Mx	TURBT	No
	BL0591F	65	pTis N0 Mx	Cystectomy	No
	BL0606F	77	pT1Nx Mx	TURBT	No
	BL0622F	63	pTis	cystectomy	
	BL0674F	54	pT1N0Mx	cystectomy	NO

*Same patient, but different surgical dates.

# Abbreviation: carbo: carboplatin; gem: gemcitabine; PTX: paclitaxel; cis: cisplatin; MVAC: Methotrexate, vinblastine, doxorubicin (Adriamycin) and cisplatin.

### Morphological fidelity between patient tumors and corresponding xenografts

**Fig 1:**
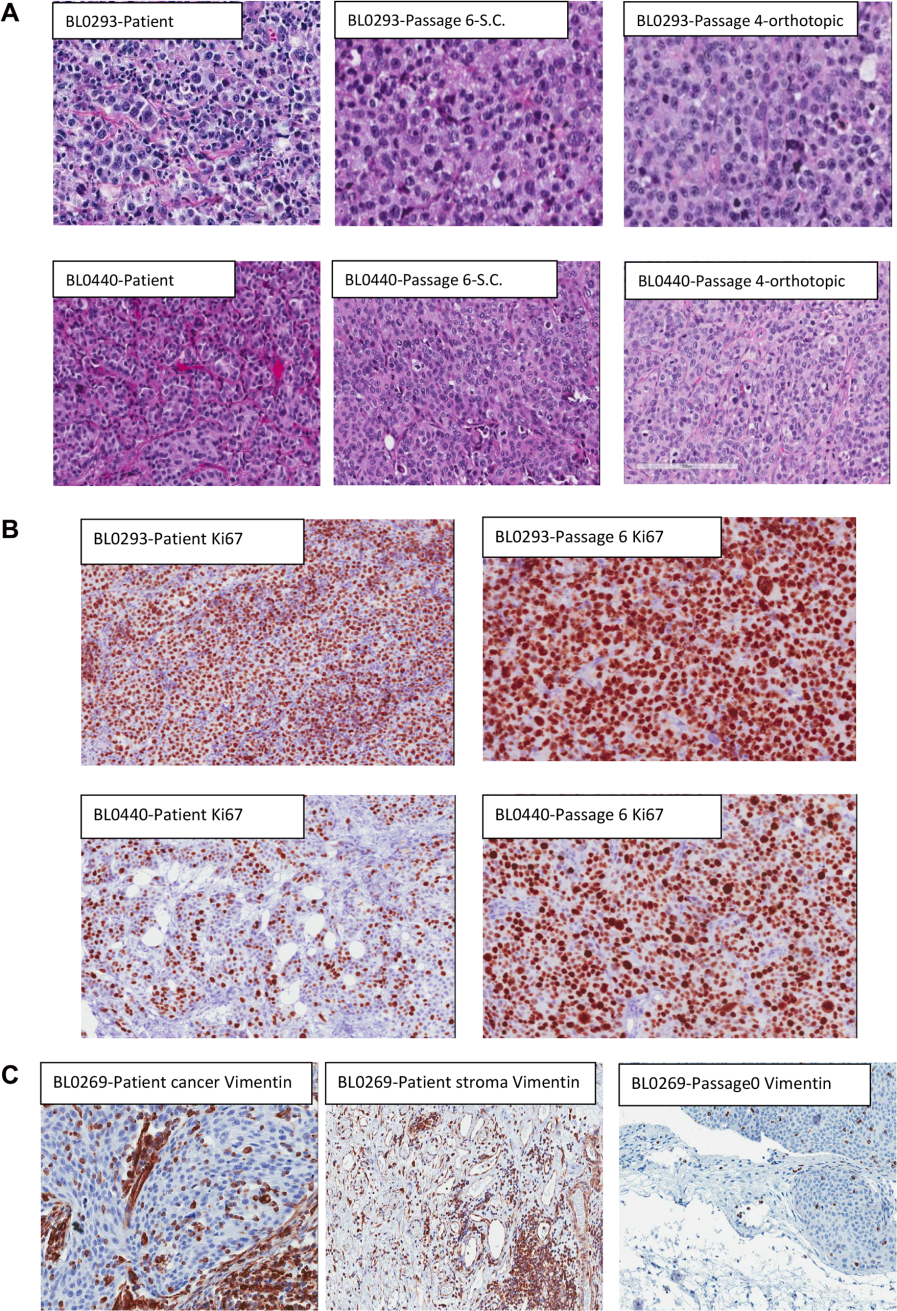
Morphology of PDX. (A) Comparison of morphology between patient specimens and PDXs, subcutaneous and orthotopic PDXs, of PDX BL0293 and BL0440. Hematoxylin and eosin stain (H & E stain) showed that cell morphology was maintained during establishment of PDX and during passaging in mice, both at the subcutaneous site (S.C., at passage 6) and at the orthotopic bladder wall (at passage 4). However, more mitotic cells were observed in PDXs, especially at passage six. (B) Staining of human Ki67. Both PDXs were stained positive with anti-human Ki67, supporting that these PDX cells were indeed of human origin. In PDX BL0440, more cells were stained positive with Ki67 at passage six PDX compared to the human bladder cancer specimen, suggesting more cells were in cell proliferation, and this finding was consistent with the observation of more mitotic cells in Panel A of H & E staining. (C) Staining of human vimentin. Some human bladder cancer cells (left panel) and stromal cells (middle panel) were stained positive for human vimentin in the patient specimens. In the PDX specimen at passage 0, only a few bladder cancer cells were stained positive for human vimentin, suggesting that the stromal cells in PDX were not derived from human stromal cells.

We compared the morphology of patient bladder tumors and corresponding PDXs at different passages. The morphological fidelity was maintained from patient tumor specimens through passage 6 **(Fig 1A)**. The morphology of subcutaneous and orthotopic bladder cancer PDXs was also maintained **(Fig 1A right panels)**. Cancer cells in PDXs were stained positive with an anti-human Ki67 antibody, confirming that the cancer cells in PDXs were indeed of human origin. Over 90% of PDX bladder cancer cells expressed Ki67, a marker of cell proliferation **(Fig 1B)**. Consistent with this finding, the PDX tumor volume doubling time was between 10–20 days. Even though PDXs were developed from inoculation of clinical specimens that included supporting human stromal cells, no stromal cells in PDXs were stained positive with an anti-human vimentin antibody, suggesting that the stromal cells in PDXs were of mouse origin **(Fig 1C)**. This is further validated by the observation of varying levels of mouse-derived sequence reads present in the next-generation sequencing (NGS) data (S2 Table). Some human bladder cancer cells and stromal cells in the clinical cancer specimens, as well as cancer cells in PDXs, were stained positive for human vimentin. As these NSG mice do not have any nature killer (NK) cells, T and B lymphocytes, no lymphocyte, by morphology, was observed in PDXs.

### Conservation of genomic variants in P0 PDX tumors

**Fig 2:**
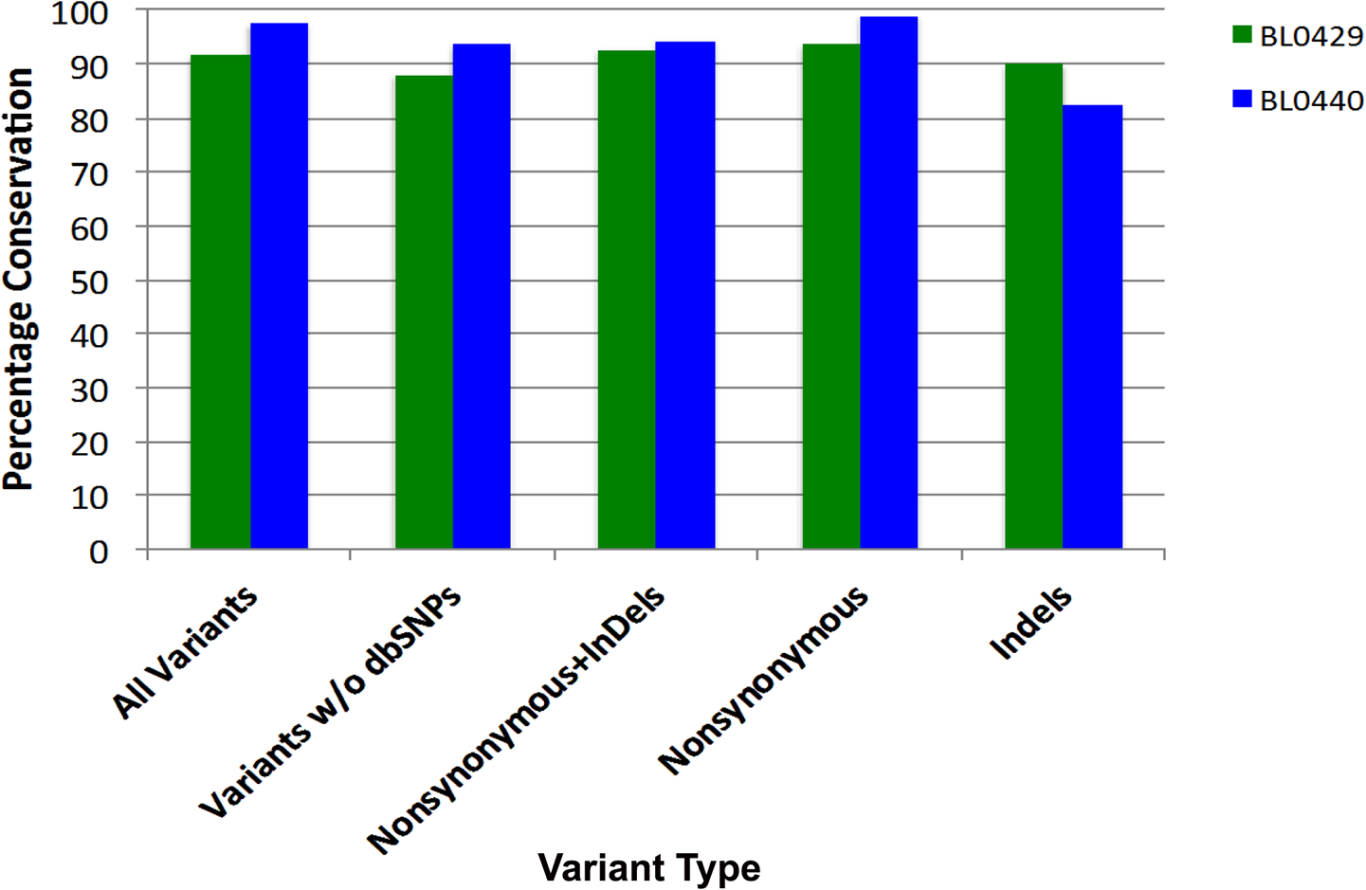
Conservation of genomic variants between patient bladder cancer specimens and PDXs. WES analysis was performed on genomic DNA samples isolated from parental patient tumors and engrafted PDX P0 tumors. WES data was filtered for variants occurring at a frequency of >0.5 and then compared between samples on the basis of variant types. The number and type of variants occurring in each parental tumor and in the P0 PDX tumor were quantified and depicted as percentage of conservation in the graph. For all variants, 91.8% and 97.6% were conserved in BL0429 and BL0440, respectively.

Comprehensive profiling of genomic aberrations in the PDXs from the first 8 patients was performed by whole exome sequencing (WES) analysis of 20,965 gene loci, encompassing 334,378 exons and spanning 71Mb of the genome (Agilent All Exon v4+UTRs capture library). To determine whether PDXs retained the genetic aberrations of parental patient cancers, WES results were compared in two PDX models, BL0429 and BL0440, at passage 0 (P0). Variants were filtered for those having a read depth of >20 and occurring at an allele frequency of ≥0.5. Of the total number variants identified in BL0429 (n = 15,653) and BL0440 (n = 16,916) patient tumors, 91.8% and 97.6% of these were conserved in the P0 tumor **(Fig 2)**. For presumed somatic mutations (*e*.*g*., nonsynonymous and indels, not found in dbSNP), a similar high level of molecular conservation was observed: 92.7% (101 of 109) and 94.4% (135 of 143) of the variants in BL0429 and BL0440, respectively **(Fig 2)**. In summary, the genomic variations present in bladder cancer patient tumors were highly conserved in the PDXs.

### Analysis of known functionally active genes and significantly mutated genes

**Fig 3:**
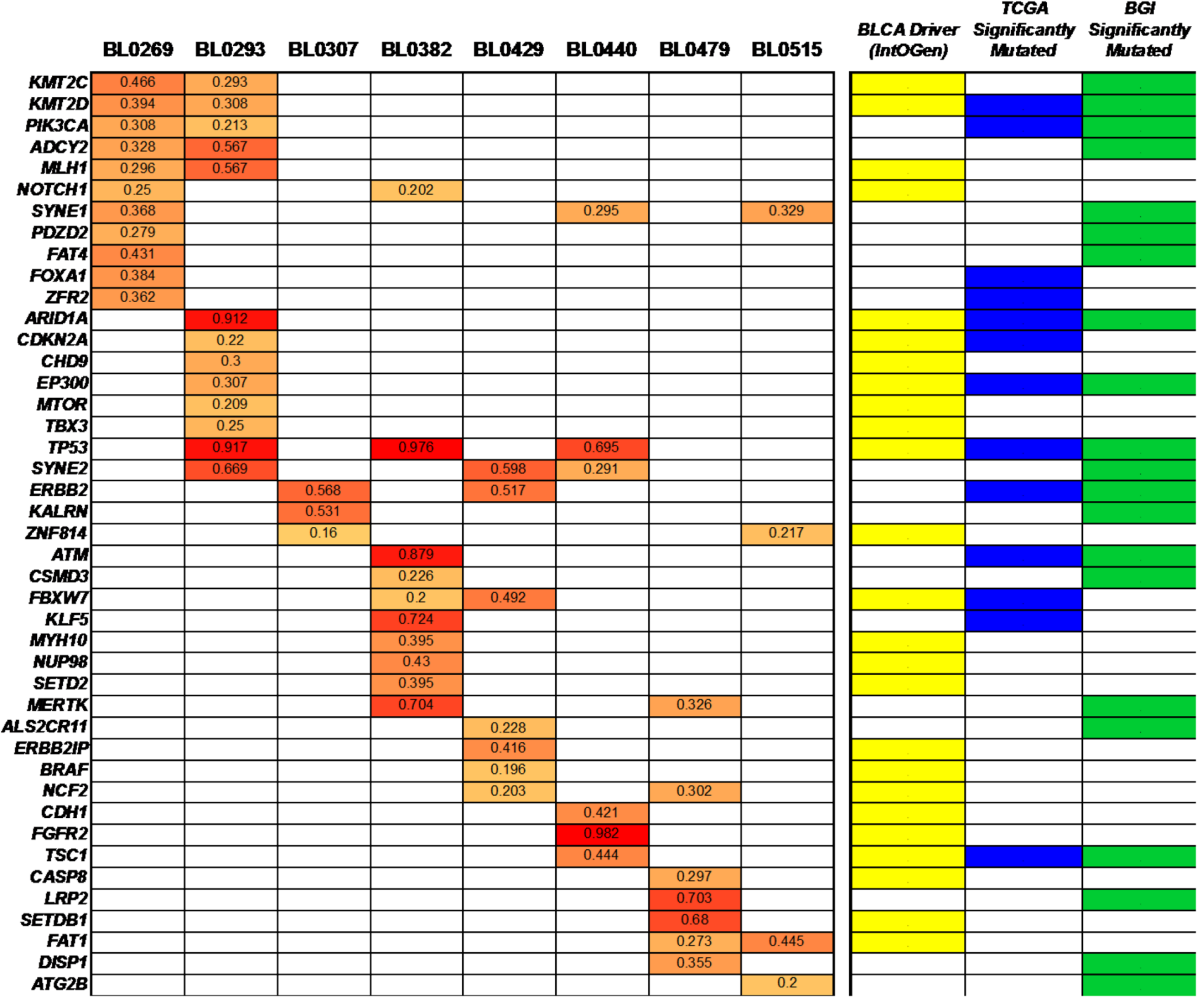
Mutation status of known bladder cancer functionally active genes and significantly mutated genes in PDXs. WES was performed on P0 tumors from each PDX model. Subsequently, the GATK was utilized for variant detection and functional annotation. The results were filtered for somatic mutations occurring in a consolidated list of 130 known bladder cancer functionally active genes [27] and significantly mutated genes [15,25,26]; these are indicated in the right side panel as *BLCA Driver (IntOGen)*, *TCGA Significantly Mutated*, or *BGI Significantly Mutated*. Somatic mutations were found in 51 different genes (indicated along the side of the table) **(S3 Table and S4 Table)**. The PDX model number is indicated across the top of the grid. Frequencies (0.3–1.0) for each mutant allele are indicated for each variant and represented by increasing color intensities.

We next assessed the mutation status of known functionally active genes and significantly mutated genes previously identified from large-scale, multi-center analyses of bladder cancers [15,25–27], many of which have demonstrated roles in tumor suppression, chromatin/chromosome dynamics, transcriptional regulation, and signal transduction. Since matched normal tissues were not included in this study, analyses were primarily directed at the identification of variants that have previously confirmed somatic status in one or more cancers according to the COSMIC and/or TCGA databases. For the 236 genes considered, a total of 71 non-synonymous single nucleotide variants (SNVs) leading to missense or nonsense mutations were identified in 51 different genes, with varying alternate allele fractions ranging from 0.160–0.982 **(Fig 3, S3 Table and S4 Table)**. While 15 genes were found to be altered in two or more tumors, only 5 mutations occurred in more than one tumor model (*ADCY2* p.V147L, *ERBB2* p.I655V, *NCF2* p.H308Q, *SYNE1* p.L885V, and *ZNF814* p.158V). Most PDXs contained 3–10 somatic mutations (in 3–10 genes), except for PDXs BL0269 and BL0293 that had 13 mutations each in 11 and 13 different genes, respectively. Notably, mutations in functionally active genes were identified in every PDX, including *ARID1A*, *ATM*, *CASP8*, *CDH1*, *KALRN*, *KMT2C*, *NCF2*, *MTOR*, *PIK3CA*, *TSC1*, and *TP53*; 6–10 mutations in bladder cancer driver genes were found in 5 of 8 models **(S4 Table)**. Integration of RNA-Seq data with these results suggested that out of the 71 mutations found, there were approximately 29 “expressed” mutations in 20 genes (*e*.*g*., *ARID1A*, *CASP8*, *KLF5*, *MLH1*, *NOTCH1*, *PIK3CA*, and *SYNE2*) that exceeded the cut-off of low/moderate transcript abundance (*i*.*e*., ≥10 FPKM or fragments per kilobase of exon per million fragments mapped) **(S5 Table)**.

### Mutational analysis of tumor suppressor pathways

**Fig 4:**
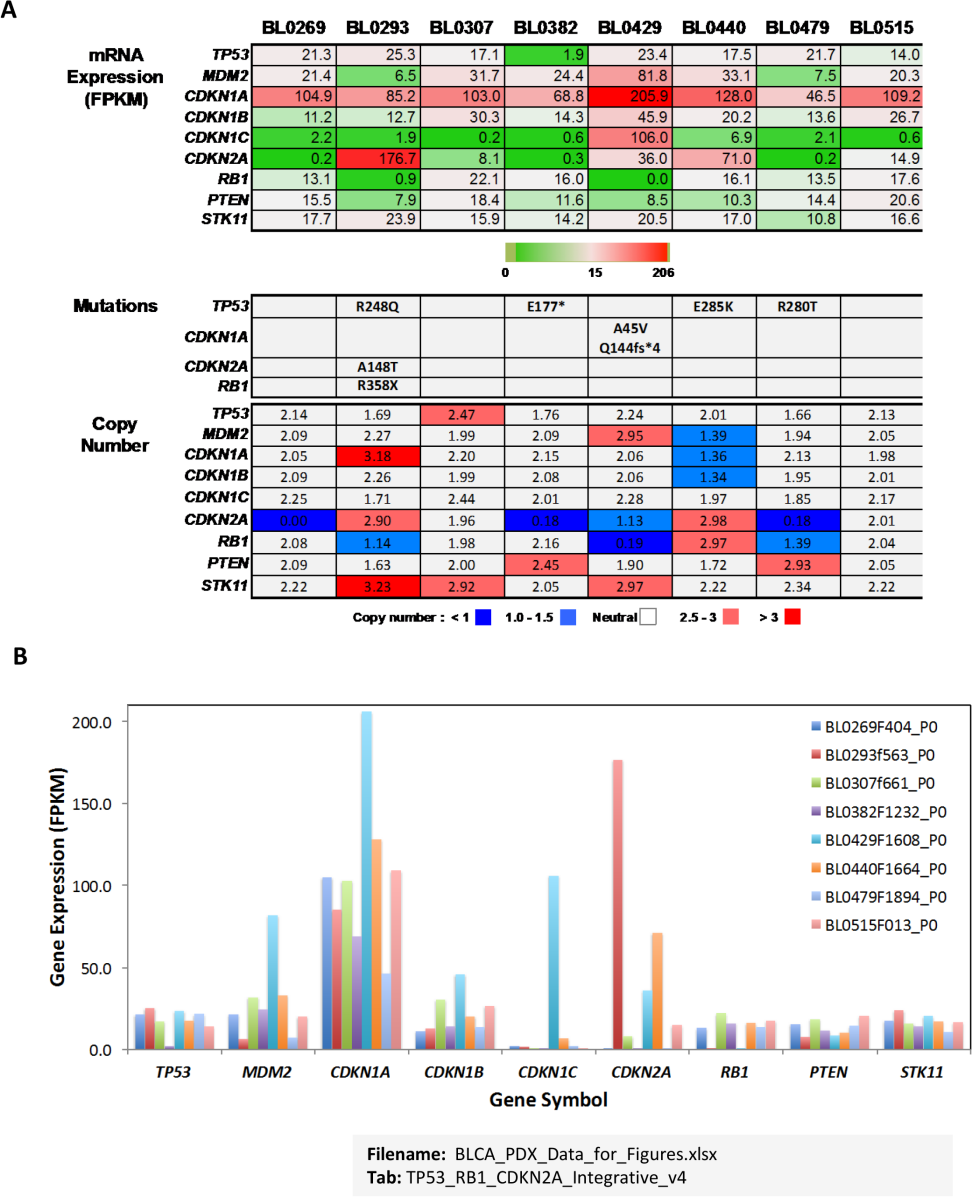
Expression levels and mutation status of tumor suppressor pathway genes. (A) Integrated analysis of *mRNA Expression*, *Mutations*, and *Copy Number* was performed for genes in the *TP53* and *RB1* tumor suppressor pathways. Gene-level expression (FPKM) values for pathway member genes are presented and relative gene expression for each gene across the panel of PDX models is indicated by the heatmap (green = lower than the median, red = greater than the median). Mutations (amino acid changes) in *TP53* and *RB1* are indicated. Gene copy numbers are presented, and variants indicated as losses (*i*.*e*., < 2) or gains (*i*.*e*., > 2) shown by darker shades of red and green, respectively. (B) Expression levels (FPKM) of tumor suppressor pathway genes in each PDX are shown in the bar graph.

Three-quarters of the bladder cancer PDXs contained aberrations in one or more of the tumor suppressor genes *TP53*, *RB1*, and *CDKN2A* **(Fig 4)**. Specifically, these included somatic mutations of *TP53* (R248Q, R280T, E177*, and E285K) and *RB1* (R358X), and copy number variants of *RB1* (in BL0293, BL0429, and BL0479) and *CDKN2A* (in BL0269, BL0382, BL0429, and BL0479). Most of the TP53 somatic mutations were heterozygous (allele frequency = 0.5) with the exception of R280T in BL0479 and E177X truncating mutation in BL0382, the latter of which was also accompanied by markedly reduced *TP53* transcript levels **(Fig 4A and 4B)**. A corresponding decrease in *MDM2* expression was observed in the BL0293 and BL0479 tumors that contain R248Q and R280T mutations in *TP53*, respectively. Copy number losses or deletions, occurring at the *RB1* and *CDKN2A* loci, were validated by comparison with the RNA-Seq data demonstrating a nearly complete absence of expression in most cases.

### Transcriptome profiling of PDX tumors

Beyond the 20 mutated genes that were found to have at least a minimal level of expression (*above*), further evaluation of gene-level expression for the entire consolidated list of 236 bladder cancer functionally active and significantly mutated genes demonstrated that 85–104 of these had moderate to very high levels of expression (FPKM = 15–2,417) in at least one PDX **(Figure A in S2 Fig, S6 Table)**. In addition, several exhibited universally high expression in the PDX models, including *AHNAK*, *BCL2L1*, *CDKN1A*, *CTNNB1*, *HRAS*, *RHOA*, and *YWHAZ*. Similarly, evaluation of the 291 high-confidence driver (HCD) genes identified via pan-cancer analysis of TCGA datasets [27], and using the same criteria for analysis as described above, demonstrated that 199 HCD genes were expressed in the panel **(Figure B in S2 Fig, S7 Table)**. As an approach to defining pathways driving the biology of PDX tumors, functional annotation clustering of the most highly expressed genes (≥50 FPKM) across the panel of PDXs was performed using DAVID bioinformatics resources tools [28]. This revealed the involvement of several fundamental cellular pathways and their molecular components, including the ubiquitin-proteasome pathway, translation factors, and metabolic enzymes (glycolysis, gluconeogenesis, oxidative phosphorylation). Notably, genes negatively regulating apoptosis were also significantly enriched (25 genes, *p* = 3.95 X 10^−06^), such as *DAD1*, *MCL1*, *SOD1*, *TPT1*, and YWHAZ, implicating the presence of a generalized survival advantage.

### PDX platform to guide molecularly targeted therapy

**Fig 5:**
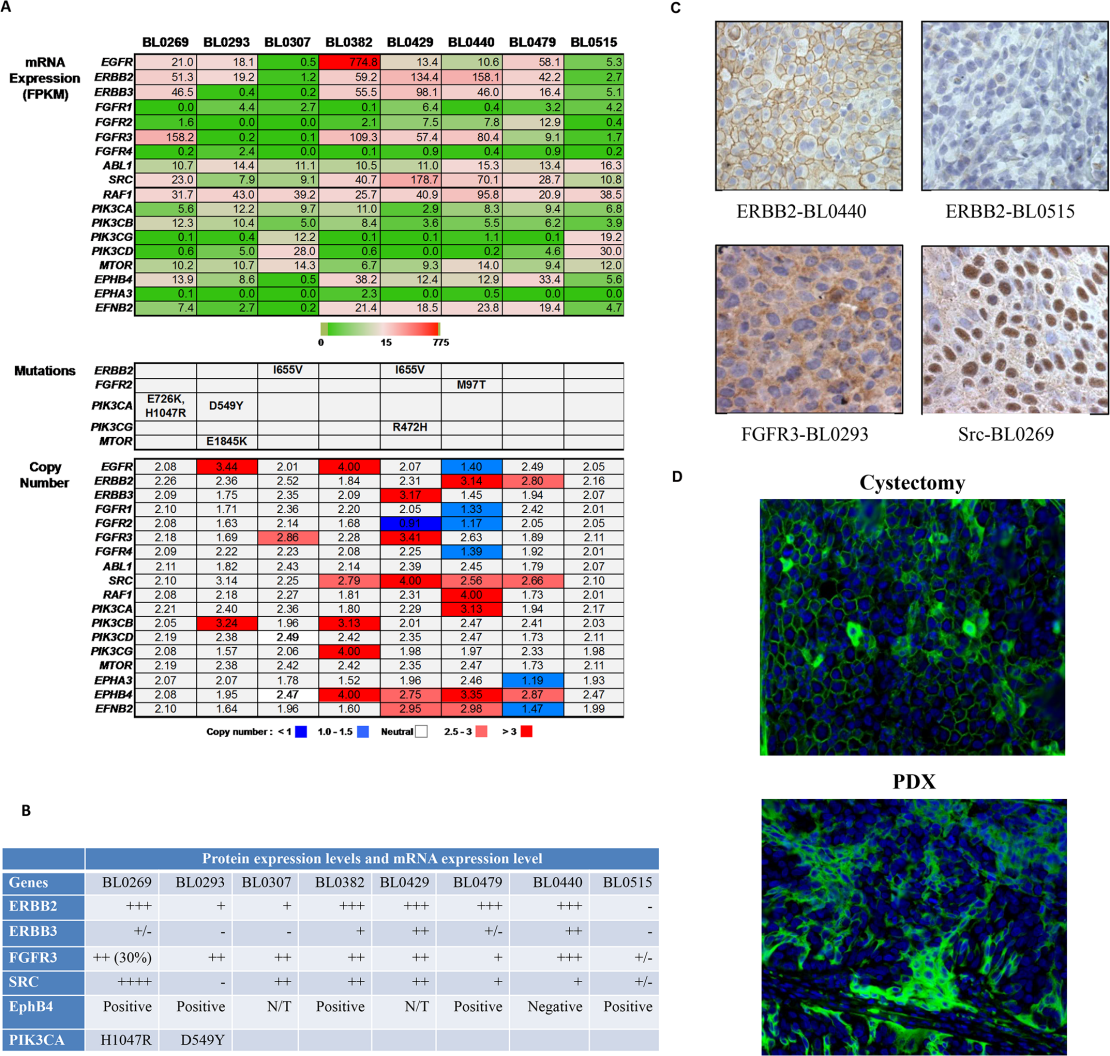
Expression status of genes encoding therapeutic targets in this study. At least 18 genes encoded direct or functional targets of signal transduction that have inhibitory agents approved by the FDA or in clinical trials. (A) Absolute expression values (FPKM), mutations and copy numbers for each of the indicated genes is shown in the bar graph. (B) Comparison of protein expression of selected target genes studied here. Protein expression levels were determined by IHC in HER2, HER3, FGFR3 and SRC, and shown in-~+++. The expression of EphB4 was determined by immunofluorescence staining and shown as positive or negative. N/T: not tested. The expression data for therapeutic targets is available in S7–S9 **Tables**. (C) Validation of target protein expression with IHC. Only selected imagings were shown. PDX BL0440 was stained 3+ for *HER2* with chicken wire pattern of positive staining at cell membrane while BL0515 was stained negative. BL0293 was stained positive for FGFR3 both on cell membrane and cytoplasm. BL0269 was stained positive for Src on cell membrane, but more on nucleus. (D) Immunofluorescence staining of tissue microarray for EphB4 of BL0293. Both patient specimen from cystectomy (left) and PDX were stained positive for EphB4.

A major goal driving the development of these PDX models was to establish a platform to help identify the correct driver mutation and its matched targeted therapy. Based on the whole exome and transcriptome sequencing data, there are many druggable targets with inhibitors that are FDA-approved or in clinical trials (**Fig 5A)** (**S7 Table, S8 Table and S9 Table**). We selected a subset of these targets to inform matched therapy testing **(S1 Fig)**: fibroblast growth factor receptor 3 (*FGFR3*), ephrin type B receptor 4 (*EphB4*), proto-oncogene tyrosine-protein kinase *Src*, human epidermal growth factor receptor -2 and 3 (*HER2/3*) and phosphatidylinositol-4,5-bisphosphate 3-kinase, catalytic subunit alpha (*PIK3CA*). Among these five targets, *FGFR3* was a particularly attractive target as it was highly expressed in four of eight PDX models (57.4–158.2 FPKM) (**Fig 5B**), and approximately 50% of primary bladder cancers [29]. The expression of these genes was confirmed by immunohistochemical (IHC) or immunofluorescence staining. In most cases, the mRNA and protein levels were correlated. As summarized in **Fig 5B**, five of the eight PDXs (BL0269, BL0382, BL0429, BL0479 and BL0440) had high *HER2* mRNA levels as determined by RNA-seq, and high (3+) *HER2* protein levels as determined by IHC staining, and manifested by the chicken wire pattern of HER2 staining on the cell membrane (**Fig 5C** upper panels); two were positive for *HER3* (BL0429 and BL0440); five were positive for *FGFR3* with a representative staining shown in **Fig 5C** left lower panel (BL0293, BL0307, BL0382, BL0429 and BL0440); and three were positive for *Src* (BL0269, BL0382 and BL0429) (**Fig 5C** right lower panel). We also found five out of eight were positive for *EphB4* (BL0269, BL0293, BL0382, BL0479 and BL0440) as determined by immunofluorescence in a tissue microarray (**Fig 5D**).

**Fig 6:**
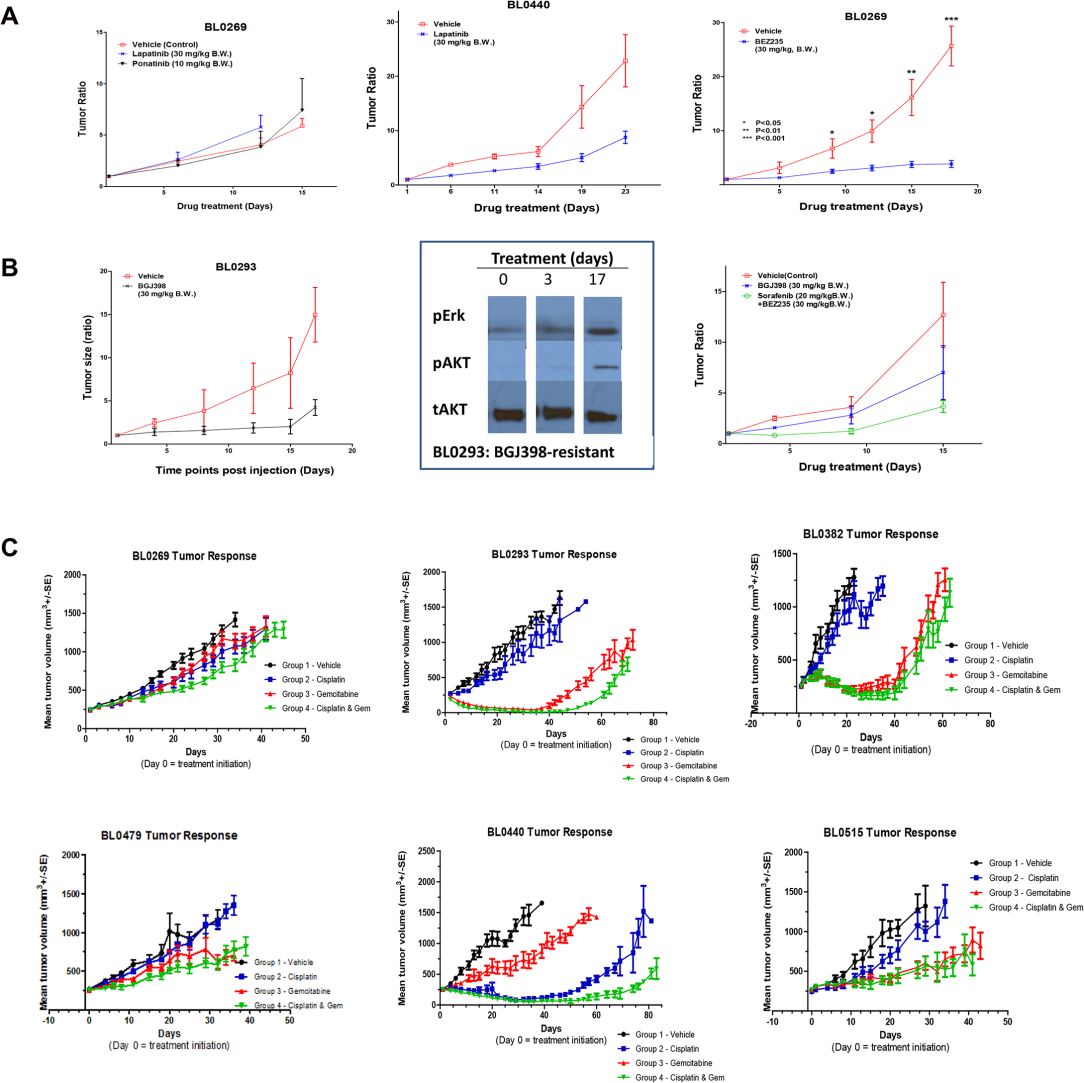
Efficacy test of molecularly guided targeted therapy matched with aberrations. (A) Efficacy studies of molecularly guided targeted therapy. BL0269 overexpressed HER2 and Src. Compared to the control group of median progression-free survival (PFS) of 13 days, a HER2 inhibitor lapatinib and a Src inhibitor ponatinib had little effect in suppressing tumor growth with a median PFS of 12 (p = 0.16) and 18 (p = 0.11) days, respectively. In contrast, lapatinib was very effective in BL0440 that expressed both HER2 and HER3 with PFS of 25.4 days versus 18.4 days in the control group (p = 0.0007). In BL0269 which also harbored a PIK3CA activation mutation H1047R, a PIK3CA inhibitor BEZ235 significantly suppressed tumor growth (p<0.0001). (B) Studies of efficacy and secondary resistance mechanisms of BGJ398 in BL0293. BL0293 overexpressed FGFR3. Compared to the control group of PFS at 9.5 days, an FGFR3 inhibitor BGJ398 significantly prolonged PFS to 18.5 days (p = 2. 61 X 10^−6^). Mice were sacrificed and PDXs were harvested (red arrows) before treatment, at Day 3 and at Day 17 (time of resistance) for western blot. Low levels of p-Akt and p-Erk at the baseline and at Day 3, suggesting low downstream signaling activity of FGFR3. Upon the development of resistance to BGJ398 at Day 17, both p-Akt and p-Erk levels increased, suggesting re-activation of the downstream signaling activity. BGJ398-resistant PDX BL0293 was re-implanted in NSG mice to form xenografts. Mice carrying BGJ398-resistant BL0293 were treated with PBS control, BGJ398, or a Raf inhibitor sorafenib plus a PIK3CA inhibitor BEZ235 combination. Compared to the BGJ398 group, treatment of BGJ398-resistant PDX with sorafenib and BEZ235 significantly prolonged PFS from 12 days to 22 days (p = 0.001). (C) Screening for effective chemotherapeutic agents. The first six PDXs were tested for sensitivity to cisplatin, gemcitabine or combination of both drugs. Only BL0440 was sensitive to cisplatin while only BL0269 and BL0479 were resistant to gemcitabine. Resistance to cisplatin could be overcome by gemcitabine, leaving four of these six PDXs sensitive to the combination therapy.

Next we determined the feasibility of using this PDX platform to address various aspects encountered during the clinical application of MTT. First we determined drug re-purposing. Lapatinib is a dual EGFR/HER2 inhibitor approved by the FDA for breast cancer. NSG mice bearing PDX BL0269 or BL0440 were treated with lapatinib or vehicle control. Both BL0269 and BL0440 expressed high levels of HER2. Yet, lapatinib was ineffective in BL0269 with a progression-free survival (PFS) of 12 days (p = 0.16), compared to 13 days in the control group (**Fig 6A**). In contrast, lapatinib was very effective (PFS of 25.4 days versus 18.4 days in the control, p = 0.007) in BL0440, which also expressed HER3 (**Fig 6A**).

Second, we used this PDX platform to screen for effective MTTs matching cancer-specific genetic aberrations. In addition to HER2, PDX BL0269 overexpressed Src and harbored an activation H1047R mutation of PIK3CA. Similar to lapatinib, a Src inhibitor ponatinib was also ineffective (PFS of 18 versus 13 days in the control. p = 0.11) (**Fig 6A**). In contrast, a PIK3CA inhibitor BEZ235 dramatically inhibited tumor growth (7.5 days of the control versus unreached over 18 days, p<0.0001).

Third, we determined the feasibility of using this PDX platform to do serial biopsies to determine the secondary resistant mechanisms, guide drug development and design of further therapy to overcome resistance. PDX BL0293 with FGFR3 overexpression was treated with the inhibitor BGJ398 that significantly prolonged PFS from the control of 9.5 to 18.5 days (*p* < 0.0001) **(Fig 6B)**. Serial biopsies revealed that, upon development of resistance, two downstream signaling pathways, MAPK/ERK and PIK3CA/AKT, were significantly activated as manifested by an increase of p-Akt and p-Erk (**Fig 6B**, **S3 Fig**). We re-transplanted and expanded the BGJ398-resistant BL0293 specimens to establish more PDXs, and found that inhibition of these MAPK/ERK and PIK3CA/AKT pathways with the combination of a PI3K/mTOR inhibitor BEZ235 and a Raf inhibitor sorafenib significantly prolonged PFS from 12 day in the control arm to 22 days in the combination arm (8 mice per treatment group, p = 0.001) **(Fig 6B)**.

Fourth, we used this PDX platform to screen for effective chemotherapy drugs. We determined the PDX sensitivity to cisplatin, gemcitabine and the combination of these two drugs. This combination is commonly used as a first-line chemotherapy in advanced bladder cancer. Of the first six PDXs we screened (10–12 mice per treatment group), five were resistant to cisplatin and two were resistant to gemcitabine (**Fig 6C**). Consistent with our previous findings that platinum agent and gemcitabine achieved additive to synergistic anti-tumor activity [30], PDXs were more sensitive to the combination than the more effective single drug as seen in BL0293 and BL0440. Chemoresistance to one drug could be overcome by the other drug, leaving four of the six PDXs sensitive to this cisplatin/gemcitabine combination.

## Discussion

Here we described the development and characterization of bladder cancer patient-derived xenografts (PDXs) with deep sequencing characterization. These PDXs maintain features of parental patient cancers as determined by both morphology and genetic aberrations.

Precision cancer medicine represents using state-of-the-art technologies to characterize the disease (cancer) and patient and then designing patient-specific therapy to explicitly target the genetic aberrations of the disease. This principle is straight forward. Several clinical trials, such as the BATTLE trial (Biomarker-integrated Approaches of Targeted Therapy for Lung Cancer Elimination) in NSCLC [31], have been proposed to determine the clinical applicability of this strategy. However, current data suggests that this approach is associated with a disappointing response rate of approximately 10% [11,12], and that the overall survival benefit is marginal even when the study included those patients whose cancers carried driver mutations such as epidermal growth factor receptor (EGFR) mutations, and who were treated with matched therapy [11]. For the FDA-approved targeted therapies, there is a wide range of response rates, from approximately 30% with trastuzumab in breast cancer with HER2/Neu overexpression and/or gene amplification [32], 60% with vemurafenib in melanoma with a BRAF mutation [33,34], to over 90% in chronic myeloid leukemia [35,36]. Moreover, when cancers do respond to targeted therapy, most will eventually develop resistance, usually within a few months [34,37,38]. Biopsies of resistant tumor specimens have been used to elucidate the mechanisms of secondary resistance [39]. Nevertheless, the paucity of patient specimens hinders further research efforts on this critical issue.

The PDX platform described here was developed with the hope it could help address some of the issues encountered with precision medicine. The essential feature of the PDX model is that each model is developed from unselected and uncultured clinical patient specimens, and shares the same genetic background as its donor patient tumor. In our PDXs, 92–97% of genetic aberrations from the original patient cancers were maintained during the development of PDXs (**Fig 2**) in addition to the morphological fidelity (**Fig 1A**). It has been shown that PDXs have relatively stable genomes without a significant accumulation of DNA structure rearrangement, but with some enrichment for PDX-unique single nucleotide variants, as shown by whole genome sequencing in primary tumors, lymphocytes and PDXs [40]. Therefore, efficacy studies conducted with PDXs will more likely reflect what happens in patient cancers. There are several studies that already showed great concordance of response between donor patient cancers and PDXs [41,42].

**Fig 7:**
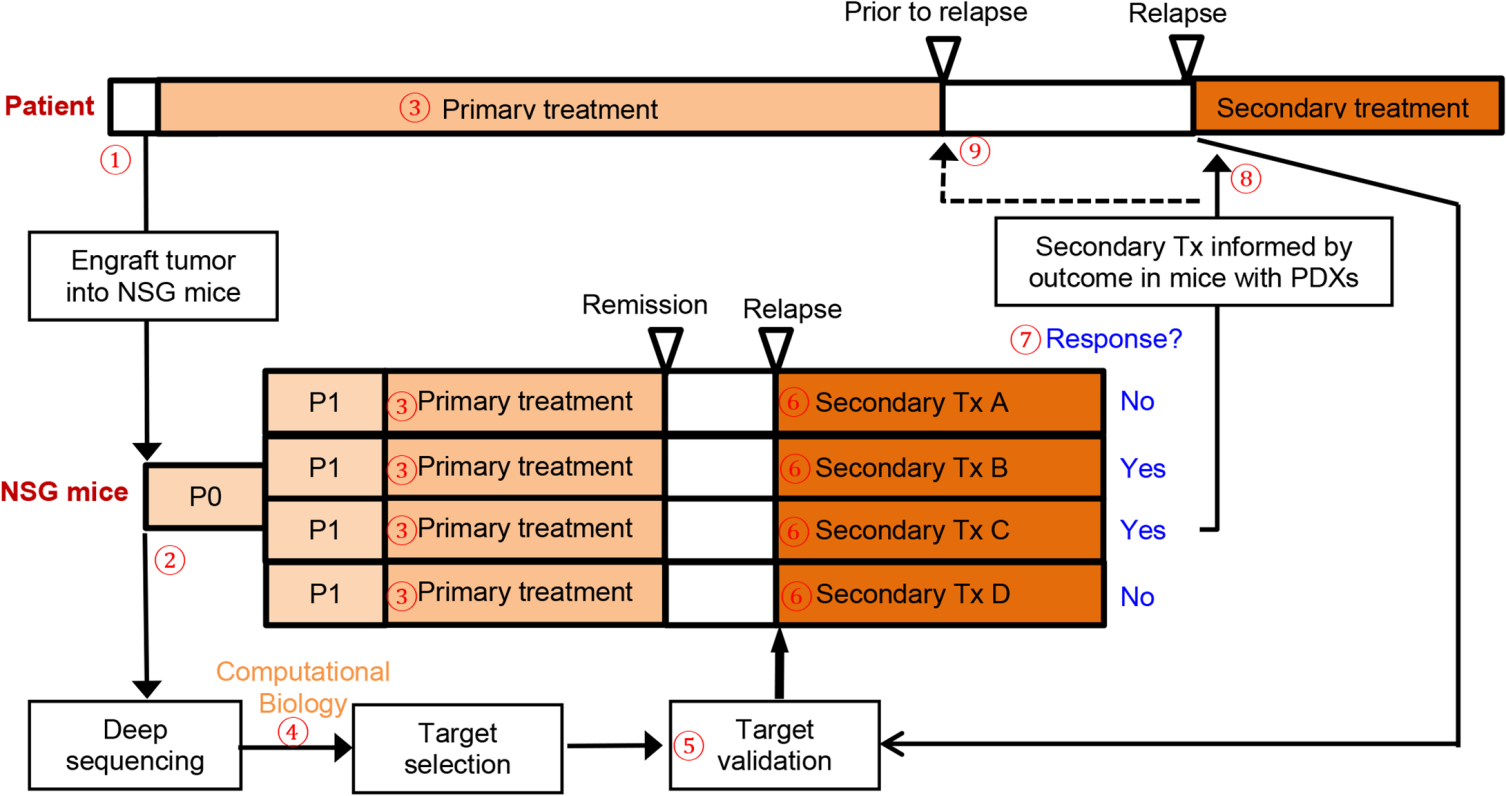
An integrated PDX para-clinical platform to improve molecularly guided targeted therapy. ① Patients with muscle-invasive or advanced bladder cancer undergo transurethral or surgery resection. Some tumor specimens are implanted into NSG mice to establish PDXs. ② When the first PDXs (Passage 0 or P0) are established, some PDX specimens are implanted to more NSG mice to establish more PDXs (P1) while some other specimens are submitted for deep sequencing, including whole genome, exome and transcriptome sequencing. ③ Patients and mice carrying P1 PDXs receive the same primary treatment. ④ Computational biology is used to identify genetic aberrations in cancer cells. ⑤ Validation tests, such as direct sequencing, western blot, IHC and immunofluorescence staining, are performed to validate the genetic aberrations as identified in Step ④. ⑥ Because of the subcutaneous location and fast tumor growth in mice, tumor relapses in PDXs will occur sooner than that in human patients. Upon relapse, mice carrying PDXs will be treated with regimens A-D targeting 4 genetic alterations identified in Steps ④ and ⑤. In addition, serial biopsies can be performed during treatment. These biopsy specimens can be studied to decipher the secondary resistance mechanisms and guide the development of further therapy to overcome resistance. ⑦ Since only some of the genetic alterations are driver ones, some medications (Drug B and C) can induce response while PDXs do not respond to some other treatments (Drug A and D). ⑧ Upon cancer relapse in human patients, biopsy can be performed to validate the existence of genetic aberrations in the relapsed cancer, then the effective drug (Drug C) matching to the genetic aberrations in human cancers is used to treat relapsed cancer in patients. ⑨ Drug C can potentially be used while patients are in remission to cure or delay cancer recurrence.

Another essential feature of the PDX platform is that many identical PDXs from a specific patient can be generated, which allows for screening of multiple targeted therapy and chemotherapy, either as single agents or combination, to select the most effective drugs. In terms of precision medicine, the major problem is that a patient’s tumor harbors multiple potential targets, ranging from several non-synonymous mutations per tumor in pediatric tumors to hundreds in colon cancer with microsatellite instability [43], with most of these genetic aberrations being passengers that are not critical for cancer cell function. In the clinical setting, time (life expectancy) and toxicity constraints only allow a limited number of different agents to be tried in each patient. Therefore, selection of the most efficacious therapy during the first few attempts will be critical in cancer treatment. The PDX allows populations of mice harboring the same tumor to be expanded to provide as many animals as are required to test the potential effectiveness of many therapeutic targets as single agents or combination, and to do so simultaneously. In terms of chemotherapy, the commonly used chemotherapeutic regimens GC (gemcitabine and cisplatin) and MVAC (methotrexate, vinblastine, doxorubicin/Adriamycin and cisplatin) are highly toxic and associated with a response rate of 50% [6]. This PDX platform may be able to determine whether cancers are sensitive to chemotherapy, and also dissect which drugs are effective (**Fig 6C**). In this study, BL0479, BL0440 and BL0515 were established from bladder cancer specimens of patients who were previously treated with chemotherapy (**Table 1**). It is possible that prior exposure to chemotherapy caused chemoresistance as seen in BL0479. Some drugs were still effective in these pre-treated PDXs, such as cisplatin in BL0440 and gemcitabine in BL0515. In addition, even when therapies are initially effective, resistance eventually develops in most cases as seen in clinic as well as in PDX studies (**Fig 6**). In these studies we report that the PDX platform allowed efficacy studies of combination therapy, either concurrent or sequential, to select the most effective therapy that in the model prolonged remission and delayed or prevented development of resistance. Furthermore, serial biopsies performed during treatment and at the time of relapse were helpful in studying the mechanisms of secondary resistance (**Fig 6B**), which is impractical in the clinical setting in patients with solid tumors. Utilizing the serial biopsy specimens, we can potentially study the resistance mechanisms and guide the design of further therapy to overcome resistance (**Fig 6B**). Based on the findings from this study and the potential clinical applications, we present an innovative model that deserves further evaluation to determine its ability to help transform the current ineffective molecularly guided targeted therapy driven by–omics technology and computational biology into an integrated model of precision medicine that combines state-of-the-art technologies with functional analysis in the PDX platform (**Fig 7**).

The PDX platform has some unique advantages for precision medicine that are not paralleled by some other cancer models, such as cancer xenografts from cell lines, and genetically engineered mouse model (GEMM). Cancer cell lines have been cultured *in vitro* for a long time, leading to the acquisition of additional genetic aberrations that differ from the original cancer. It has been shown that, even after a few generations, there was a great irreversible genetic divergence between a primary tumor and the cell line derived from that tumor [44]. Hence, it is not surprising that prediction models for drug response that were based on the genetic information of cell lines, such as the Genomics of Drug Sensitivity in Cancer [45] and the Cancer Cell Line Encyclopedia [46], frequently fail to predict drug efficacy in the clinic [7]. In terms of GEMM, several techniques are used to modify the murine gene expression, ranging from germline transgenesis, gene knockout or mutation, to more sophisticated temporal and spatial control of gene expression (reviewed [47]). Cancers usually develop within a few months to 1–2 years in these mice. Even though GEMMs manifest some aspects of oncogenesis of human cancer, cancers developed in GEMM are relatively genetically homogenous. In contrast, many human cancers develop after years of exposure to carcinogens from smoking and environment, and harbor multiple and diversified genetic alterations. For instance, in bladder cancer, about two thirds of the cancers are attributed to smoking. As shown in **Figs 3, 4 and 5**, each cancer carries a unique spectrum of genetic alterations. Therefore, preclinical studies in GEMMs may not meet the needs to design patient-specific therapeutic regimen to target the unique genetic alterations.

There are several disadvantages associated with the PDX platform [48] that must be considered when drawing conclusions from their use. One drawback is that not all tumors establish a xenograft, which means that PDX platform is not representative of the patient populations. It usually takes 4–5 months to establish the first PDX (P0) but only 2–5 weeks for subsequent passaging of PDXs. This long time lapse makes the PDX platform more of a research tool (vs avatar), especially in cancers with rapid disease progression and short patient survival, as reported in non-small cell lung cancer and pancreatic cancer (reviewed [49,50]) [51]. However should the PDX model work as hoped, the relatively slow rate of disease progression makes bladder cancer one of the best candidate cancers in translating preclinical findings in PDXs into clinical applications. Additional limitations include replacement of the stromal components with the mouse stroma (Fig 1) and lack of immune response in immunodeficient mice. Our future research efforts will focus on overcoming these limitations where possible and further defining the limits of translating PDX results to patient outcome.

If fully validated, the PDX platform may have clinical applications, including: screening of multiple therapeutic agents simultaneously to select the most efficacious drugs or drug combination for patient treatment; deciphering the mechanisms of primary resistance; and developing biomarkers for patient selection in clinical decision making. These will all contribute towards turning molecularly targeted therapy into precision cancer medicine.

In conclusion, we have developed and characterized a patient-derived bladder cancer xenograft model platform that may prove useful for screening of effective drugs or drug combinations, and to study mechanisms of resistance. The use of this platform may accelerate drug discovery leading to improve treatments for bladder cancer.

## Supporting Information

S1 Fig Target genes and matched targeted therapies. (DOCX)

S2 Fig Expression analysis of bladder cancer functionally active genes and significantly mutated genes. (PPTX)

S3 Fig FGFR3 downstream signaling pathway and activity. (DOCX)

S1 Table Summary of NGS read statistics. (XLSX)

S2 Table Evaluation of infiltration of PDX tumors by host mouse cells. (XLSX)

S3 Table Mutations of BLCA Significantly Mutated Genes Identified in BLCA PDX Models. (XLSX)

S4 Table Summary data for mutations identified in significantly mutated genes in BLCA PDX models. (XLSX)

S5 Table Integrated Mutation and Expression Data for Significantly Mutated Genes Identified in BLCA PDX Models. (XLSX)

S6 Table Expression Levels of BLCA Driver and Significantly Mutated Genes—Identified by TCGA, IntOGen, and BGI Analyses. (XLSX)

S7 Table Gene-level expression of high-confidence driver genes in P0 PDX tumors. (XLSX)

S8 Table Expression of Genes Encoding Therapeutic Targets of Agents that are Approved, in Clinical Trials, and/or listed as a COSMIC Compound. (XLSX)

S9 Table Gene Expression Levels of Protein Tyrosine Kinases. (XLSX)
