# Supplementary material for: Development and Characterization of Bladder Cancer Patient-Derived Xenografts for Molecularly Guided Targeted Therapy
Source: PLoS One. 2015 Aug 13;10(8):e0134346. doi: 10.1371/journal.pone.0134346 (PMC4535951; doi:10.1371/journal.pone.0134346)
Supplement: S1 Fig — (DOCX) [file pone.0134346.s001.docx]

| Genes and corresponding targeted therapeutic agents | | | | | |
| --- | --- | --- | --- | --- | --- |
| Genes | **Drugs** | **Dose**  **(mg/kg)** | **Route** | **Schedule** | **Current status** |
| ERBB2 | Lapatinib | 30 | oral | Twice daily | Approved for breast cancer |
| ERBB3 |  |  |  |  |  |
| FGFR3 | BGJ398 | 30 | oral | Once daily | Phase II |
| SRC | ponatinib | 10 | oral | Once daily | Approved for CML |
| EphB4 | sEphB4-HSA | 50 | s.c. | 3 times weekly | Phase I |
| PIK3CA | BEZ235 | 30 | i.v. | Twice weekly | Phase I |

**SI-I. Target genes and matched targeted therapies**

Target genes and their corresponding targeted therapy drugs, dose, route of administration in the efficacy studies in NSG mice carrying PDXs, as well as the current status in drug development
