# Supplementary material for: Development and Characterization of Bladder Cancer Patient-Derived Xenografts for Molecularly Guided Targeted Therapy
Source: PLoS One. 2015 Aug 13;10(8):e0134346. doi: 10.1371/journal.pone.0134346 (PMC4535951; doi:10.1371/journal.pone.0134346)
Supplement: S2 Fig — (PPTX) [file pone.0134346.s002.pptx]

## Slide 1
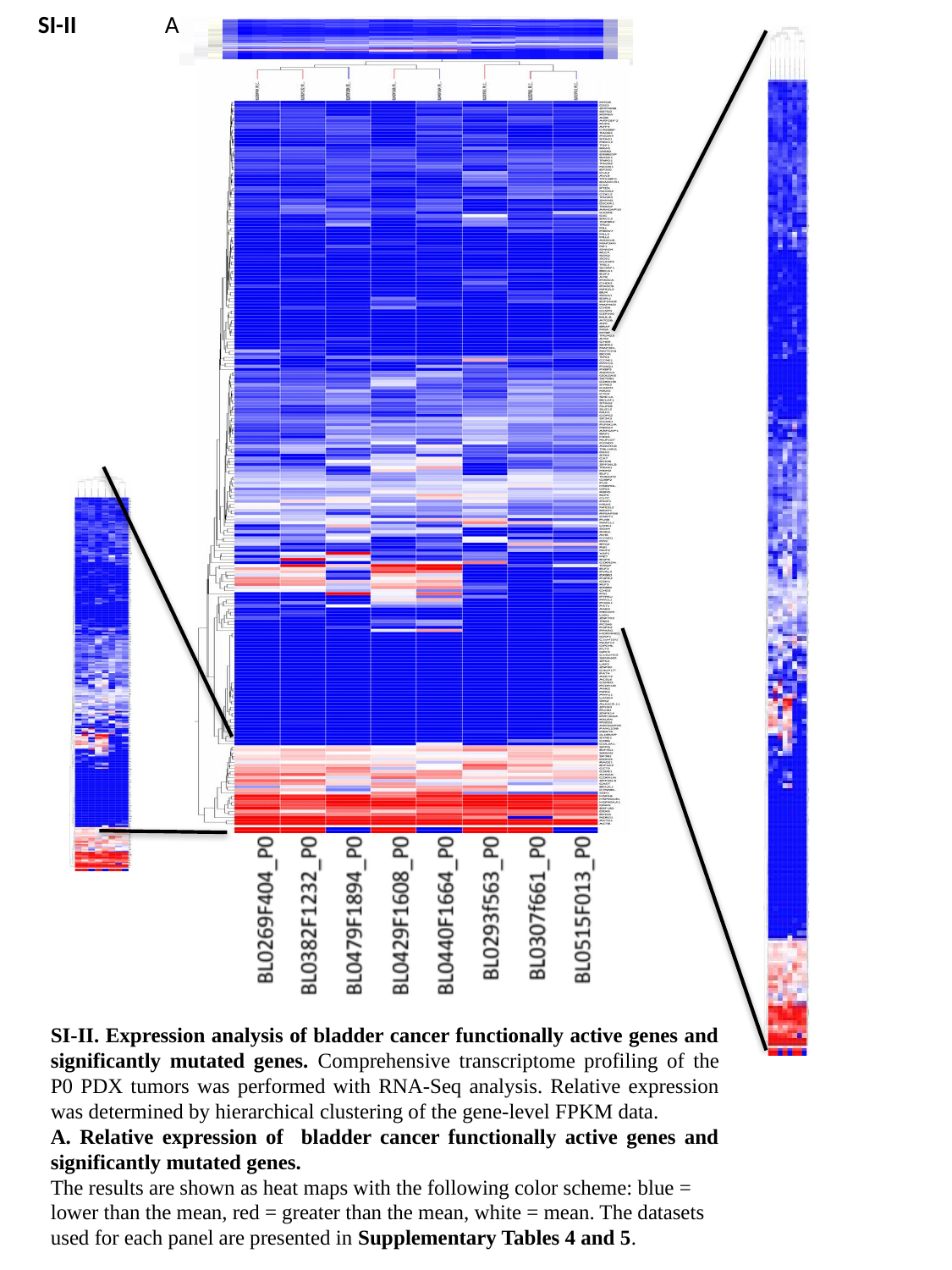

# SI-II	A
SI-II. Expression analysis of bladder cancer functionally active genes and significantly mutated genes. Comprehensive transcriptome profiling of the P0 PDX tumors was performed with RNA-Seq analysis. Relative expression was determined by hierarchical clustering of the gene-level FPKM data.
A. Relative expression of bladder cancer functionally active genes and significantly mutated genes.
The results are shown as heat maps with the following color scheme: blue = lower than the mean, red = greater than the mean, white = mean. The datasets used for each panel are presented in Supplementary Tables 4 and 5.

## Slide 2
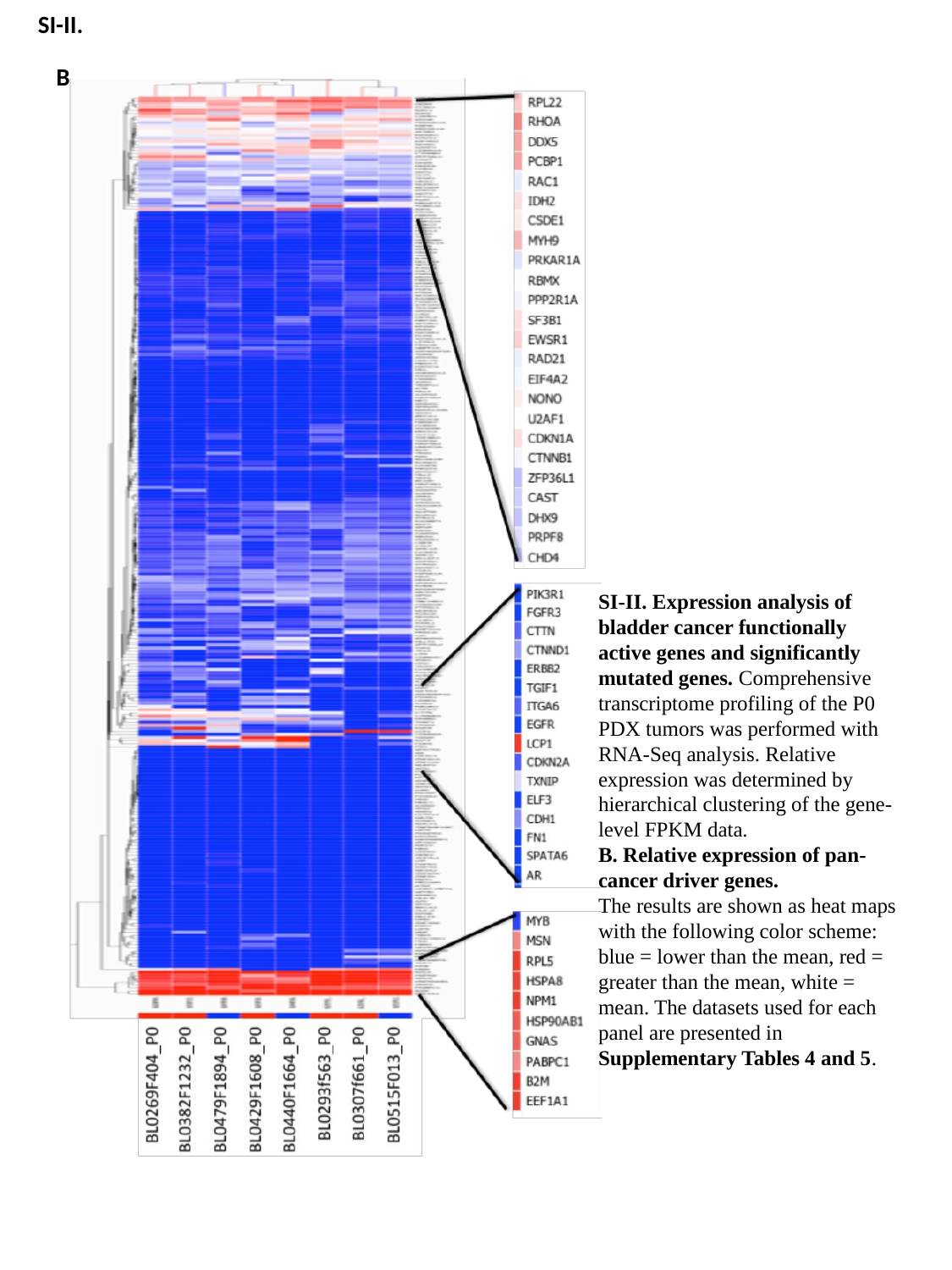

# SI-II.
B
SI-II. Expression analysis of bladder cancer functionally active genes and significantly mutated genes. Comprehensive transcriptome profiling of the P0 PDX tumors was performed with RNA-Seq analysis. Relative expression was determined by hierarchical clustering of the gene-level FPKM data.
B. Relative expression of pan-cancer driver genes.
The results are shown as heat maps with the following color scheme: blue = lower than the mean, red = greater than the mean, white = mean. The datasets used for each panel are presented in Supplementary Tables 4 and 5.
