# Supplementary material for: Development and Characterization of Bladder Cancer Patient-Derived Xenografts for Molecularly Guided Targeted Therapy
Source: PLoS One. 2015 Aug 13;10(8):e0134346. doi: 10.1371/journal.pone.0134346 (PMC4535951; doi:10.1371/journal.pone.0134346)

**SI-III. FGFR3 downstream signaling pathway and activity**

1. Simplified diagram of the cell surface tyrosine kinase receptor downstream signaling pathways. There are three main downstream signaling pathways: the RAS-RAF-MEK-ERK pathway, PI3K-AKT pathway and STATs pathway. As shown in Panel B, the STATs pathway is not active in bladder cancer as no phosphorylated STATs was detected.


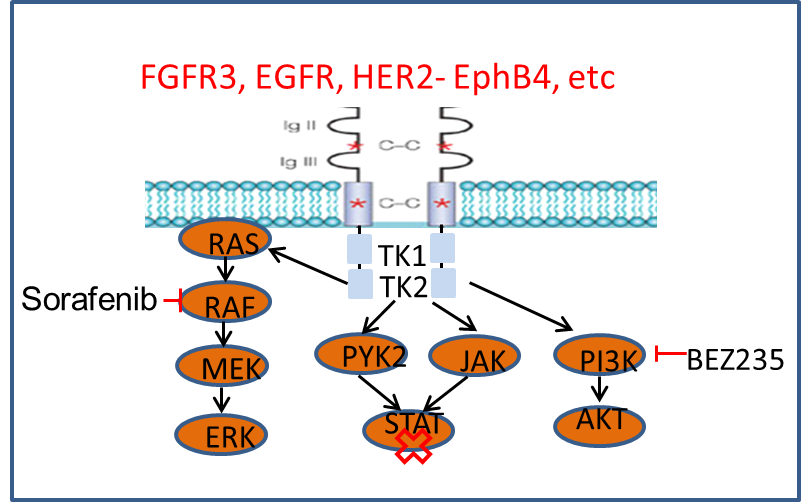


1. Western blot to determine the downstream signaling pathway activity. Low phosphorylated AKT (p-AKT) and phosphorylated ERK (p-ERK) were detected at 3 days after treatment with the FGFR inhibitor BGJ398, suggesting both downstream signaling pathways were effectively inhibited. Upon development of resistance to BGJ398 at Day 17 of treatment, p-AKT and p-ERK were up-regulated, suggesting that these two downstream signaling pathways were re-activated. No significant phosphorylated STAT3 were detected at all time points, suggesting that this pathway is not active.

BL0293-BGJ398 Treatment

p-AKT (Ser473)

AKT1

p-ERK (Tyr204)

ERK

p-STAT3 (Y705)

STAT3

Tubulin


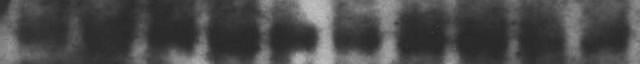

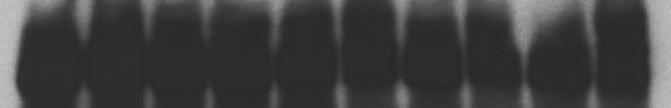

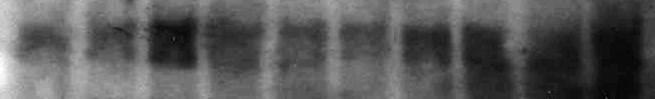

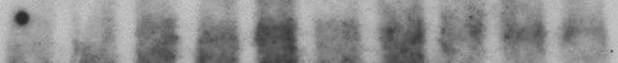

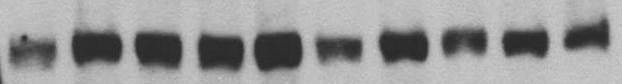


0 day

3 day

17 day


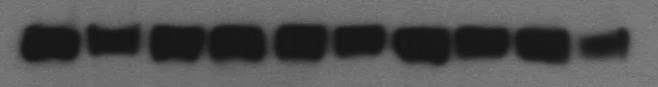

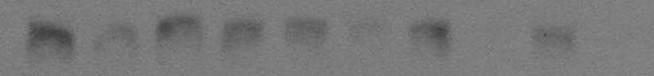

Supplement: S3 Fig — (DOCX) [file pone.0134346.s003.docx]
